# Phenotypic plasticity determines differences between the skulls of tigers from mainland Asia

**DOI:** 10.1098/rsos.220697

**Published:** 2022-11-30

**Authors:** David M. Cooper, Nobuyuki Yamaguchi, David W. Macdonald, Olga G. Nanova, Viktor G. Yudin, Andrew J. Dugmore, Andrew C. Kitchener

**Affiliations:** ^1^ Department of Natural Sciences, National Museums Scotland, Edinburgh EH1 1JF, UK; ^2^ Institute of Geography, School of Geosciences, University of Edinburgh, Edinburgh EH8 9YL, UK; ^3^ Institute of Tropical Biodiversity and Sustainable Development, University Malaysia Terengganu, Kuala Nerus, Terengganu 21030, Malaysia; ^4^ Wildlife Conservation Research Unit, Department of Zoology, University of Oxford, The Recanti-Kaplan Centre, Tubney House, Abingdon Road, Abingdon, Oxfordshire OX13 5QL, UK; ^5^ Zoological Museum, M.V. Lomonosov Moscow State University, Bolshaya Nikitskaya 2, Moscow 119991, Russia; ^6^ Federal Scientific Centre for the Biodiversity of Terrestrial Biota of East Asia, Far Eastern Branch, Russian Academy of Sciences, Vladivostok, Primorskij kraj, Russia; ^7^ Human Ecodynamics Research Centerand Doctoral Program in Anthropology, City University of New York (CUNY), NY 10017, USA

**Keywords:** phenotypic plasticity, *Panthera tigris*, morphometrics, taxonomy, captive/wild comparison, zoo welfare

## Abstract

Tiger subspecific taxonomy is controversial because of morphological and genetic variation found between now fragmented populations, yet the extent to which phenotypic plasticity or genetic variation affects phenotypes of putative tiger subspecies has not been explicitly addressed. In order to assess the role of phenotypic plasticity in determining skull variation, we compared skull morphology among continental tigers from zoos and the wild. In turn, we examine continental tiger skulls from across their wild range, to evaluate how the different environmental conditions experienced by individuals in the wild can influence morphological variation. Fifty-seven measurements from 172 specimens were used to analyse size and shape differences among wild and captive continental tiger skulls. Captive specimens have broader skulls, and shorter rostral depths and mandible heights than wild specimens. In addition, sagittal crest size is larger in wild Amur tigers compared with those from captivity, and it is larger in wild Amur tigers compared with other wild continental tigers. The degree of phenotypic plasticity shown by the sagittal crest, skull width and rostral height suggests that the distinctive shape of Amur tiger skulls compared with that of other continental tigers is mostly a phenotypically plastic response to differences in their environments.

## 1. Introduction

Morphological studies provide perspectives on genetic expression and the functional diversity of different clades and clines [1,2]. Alongside molecular and biogeographical lines of enquiry, they are also used to determine the taxonomic status of species and subspecies [3]. While morphological differences are known to exist between captive and wild vertebrates [4–10], the potential influence of an individual's life history is rarely accounted for in population-level and continental-scale morphological studies of wild mammals. The aim of this paper is to determine how phenotypic plasticity can affect the skull morphology of a large mammalian species, the continental tiger (*Panthera tigris tigris*), using captive–wild and wild–wild comparisons. The basis for this comparison is that the genetics of wild and captive tigers are the same, but their environments, including diets, are different, so that we can explore the phenotypic response to those environmental differences. In doing so, we can better understand the extent to which morphological variation in the natural environment is determined by an individual's environmental interactions and life history, or by long-term evolutionary processes.

The number of recognized tiger subspecies varies between two and nine (six extant) and remains contentious, mainly because of differences in the interpretation of data [11–13]. However, the most comprehensive study recognized only two subspecies, the continental tiger (*Panthera tigris tigris*) and the Sunda Island tiger (*Panthera tigris sondaica*) based on genetic, biogeographical and morphological data [11,14,15]. Skull morphology has been used to both differentiate [16–18] or show similarities [11,17–19] between populations of the tiger using statistical techniques applied to skull metrics. For example, the sagittal crest has been observed to be more developed in wild Amur and Caspian tigers than in other mainland tigers [17,20,21], yet there is a lack of explicit analytical consideration of the driving factors of morphological variation. It is unclear whether there are fixed genetic differences between the morphologies of putative tiger subspecies as a result of natural selection, or whether these differences are phenotypic responses to varying environments. The continental tiger represents up to six genetic clades [12,22–24] that until recently had a contiguous range across continental Eurasia during the Late Pleistocene and early Holocene [14], and now covers latitudes from +1^o^ to +48^o^ N. Its distribution ranges from equatorial to boreal forests with vast differences in climate and prey communities. The broad geographical range, known genetic structure, and availability of specimens from both captivity and the wild make the continental tiger well suited to a study that attempts to disentangle phenotypic plasticity from genetic expression. We do not include tigers from the Sunda Islands (*Panthera tigris sondaica*), to simplify our analyses by reducing potential confounding sources of variation (island founder effects, island rule of body size [25], and long-term vicariance from continental tigers [14,26]).

Phenotypic variation can result from multiple mechanisms operating at different spatial and temporal scales [27]. Differences in prey size and killing techniques may dictate differing morphological traits between species of wild felid due to long-term evolutionary processes [28,29]. The killing strategy of big cats varies depending on the species and its prey size. Typically big cats, *Panthera* spp., kill smaller prey with a nape bite, which severs the spinal cord, or a throat or muzzle bite, which causes the suffocation of larger prey [29,30]. Availability of different prey sizes varies throughout widespread species' ranges, so that the development of skulls and mandibles may be affected by mechanical forces acting on them as a result of the different kinds of prey and killing bites used, potentially resulting in variation in skull and mandible shape. Alternatively, the functional differences between species, and populations of the same species, may simply arise from the genetic fixation of phenotypic variations through natural selection acting on different lineages and clades and it may not be affected by adaptive processes such as those relating to bite force, killing strategy or the mechanical properties of food items under different environmental conditions [30]; or it may be a combination of phenotypic and genetic factors.

Skull shape differences among subspecies may also result from population bottlenecks and genetic drift, which can lead to accelerated morphological and behavioural evolution [1], and thus morphological differences within a species may be the result of recent evolutionary and demographic history. Morphological evolution in mammals can occur very rapidly within small isolated populations, when they are presented with altered environmental conditions over periods ranging from a few decades to several thousand years [31–34], and these rapid morphological changes can occur without the genetic signal of long-term vicariance [35]. Rapid evolutionary processes are especially important when considering isolated current populations of the continental tiger within the Russian Far East, commonly known as the Amur tiger, which have had reduced contact with other populations since before historic times [14].

The most localized mechanism for morphological variation occurs due to the phenotypic plasticity of individuals within their respective environments. Genes do not directly encode bone shape beyond the patterning of the embryo [36], and bone is a phenotypically plastic tissue, which is capable of large changes in size and shape in response to a multitude of influences [37]. While well documented through studies of the morphological differences between captive and wild specimens of the tiger [8,9], phenotypic plasticity in skull morphology is less well understood across wild populations, where differences may occur due to variation in prey size, type and abundance, and the effects these have on mastication, killing technique and nutrition. The phenotypic plasticity of bone may provide an ecological advantage by allowing for a wide range of sizes that a morphotype can occupy in response to local environmental conditions [38]. In grizzly bears (*Ursus arctos*), meat consumption has been linked to skull parameters as indices of nutrition during stages of development [39]. As cats are obligate carnivores, similar patterns are likely in the skulls of the continental tiger. A carnivorous diet, which involves the biting and chewing of tough skin, muscle, connective tissues and hard bone, results in considerable forces acting on the teeth and skull during killing and mastication. Therefore, it is probable that the environmental conditions, such as prey size, type and abundance, present during the development of an individual will affect skull size or shape by way of a phenotypically plastic response to killing and consuming prey [2,40,41].

The skulls of captive big cats are wider, with greater rostral and mastoid breadths, broader zygomatic arch widths, and broader mandibles than those of wild individuals [8,42,43]. The mechanical influences of diet on biting and chewing have been hypothesized as the driving cause of morphological differences between wild and captive carnivorans [4,7,42]. Wild big cats have been shown to possess greater skull dimensions in areas that experience higher forces compared with captive individuals, which are not subjected to such forces as a result of softer foods, leading to significant morphological differentiation [43]. Beyond the mechanical properties of diet, it is possible that captive diets vary from the wild in their nutritional properties. Protein and fat digestibility can vary depending on the type of processing applied to food [44], and between dietary items [45].

An understanding of the plasticity of skull morphology due to the very different environmental conditions and diets of captivity and the wild allows us to determine the extent to which the environmental conditions experienced by individuals, as opposed to evolutionary history, influence skull morphology in wild populations. This is important because putative subspecies across the geographical ranges of species are in part identified through morphological studies, which assume a dominant role of evolutionary history in determining differences in skull size and shape. Identifying how phenotypic plasticity influences skull shape enables better assessment of the extent to which environment affects skull morphology in wild populations. While genetic studies have superseded morphological studies as the customary determinant of phylogeny, whole-genome studies are still at an early stage and only explain a few drivers of morphology explicitly in terms of gene expression [12], and so trying to determine variation by other means is still of importance. Boundaries of subspecies and evolutionarily significant units, as recognized and defined by morphological differences, influence conservation management, and so have a real, practical importance for the future of threatened species.

This paper is based on significantly bigger datasets than previous craniometric studies that compare captive and wild specimens. It includes measurements from both wild and captive continental tigers representing all previously reported continental subspecies. Our analysis is structured around three research questions that use the profound differences between the environmental conditions of wild and captive continental tigers to understand variation between wild populations.

### 1.1. Does captivity status account for skull variation in the continental tiger?

Using a captive–wild comparison, we identify patterns of skull variation that occur due to a plastic response of the skull to the vastly different environmental conditions of captivity compared with the wild.

### 1.2. Do known phylogenetic clades account for skull variation in the continental tiger?

The effects of recent and long-term evolutionary patterns upon the continental tiger are assessed in relation to recent phylogenetic clades [12].

### 1.3. Do patterns of variation between wild populations differ from patterns of variation between captive and wild specimens?

By comparing variation between wild populations of different putative subspecies with variation caused by phenotypic plasticity (i.e. due to captivity), we ascertain whether wild variation has probably occurred from evolutionary processes or genetic drift, or from plastic changes due to environmental differences between populations.

## 2. Materials and methods

### 2.1. Data

This paper examines linear cranial measurements of 172 continental tigers from museum collections across Europe, from both captivity and from wild populations. The captive specimens are individuals with known provenance with individual global studbook numbers, which had been born and died in zoos. Captive breeding programmes aim to equalize the founder contributions to the gene pool, thereby minimizing inadvertent genetic selection for particular traits. Captive tigers are likely to represent a random sample of wild populations due to a large number of founder individuals from over a century of continual influxes of animals from the wild from a broad geographical background [22]. Subadults were not included in the analysis, because skull development in these individuals was still occurring at the time of death [46]. Subadults are defined by the basioccipital-basisphenoid suture, and/or frontal suture still being unfused [47].

Seventy-four linear measurements and cranial volume were recorded for each specimen, following Barnett *et al.* [47] (see electronic supplementary material, table S1). As a quality-control measure, five skulls were selected for three repeat measurements on different days, to test for intra-observer measurement error. Nineteen measurements were removed from further analysis where mean coefficient of variation was above 1%, found either through this study or from Barnett *et al.* [47] (electronic supplementary material, table S1 and appendix S2). The final dataset comprises 56 measurements (electronic supplementary material, table S1).

Within our dataset, 9% of missing data affects 38% of specimens. To enable the use of principal component analysis (PCA), which requires no missing data, we use multiple imputation by chained equations to maximize the number of specimens available for analysis (see electronic supplementary material, appendix S2)

### 2.2. Data scaling and shape principal component analysis

Size and shape were considered separately to differentiate influences of allometric scaling [17,18]. The analysis undertaken here follows the methodology of Baur & Leuenberger [48], to log-transform and centre the data, so that measurements are independent of size (although not independent of allometry [49]). By scaling the data in this way, the effects of isometric size can be assessed in isolation from shape and allometry. A PCA was performed upon the scaled variables to create shape principal components (sPCs). Isometric size (isosize) of individuals was calculated as the geometric mean of all variables [48,50]. The relationships between the scaled variables, sPCs and isosize were examined in relation to captivity status.

### 2.3. Data partitioning

Sexes were analysed separately, because big cats exhibit strong sexual dimorphism [7,18,46]. The available data were split into five genetic clades for the tiger within continental Eurasia [12], because geographical variation in both skull size and shape has been found in previous studies of the tiger [11,16,17,19]. When analysing each of these subsets, the data were rescaled before repeating the analyses. By splitting the data by sex and into groups of similar geographical origin, independent datasets were created to corroborate patterns found between captive and wild specimens.

### 2.4. *t*-Tests

Standard *t*-tests were used to highlight variables that were significantly different between captive and wild specimens, and highlight variable differences between wild populations that show morphological differentiation in shape PCA plots. Owing to the large number of measurements, a Bonferroni correction was applied by dividing the standard *p*-value of 0.05 by the number of tested variables. The standard 0.05 value is also displayed to show variables, which may differ significantly. By highlighting measurements that differ significantly, overall patterns of variation are visualized upon diagrams of skull measurements.

## 3. Results

**Figure 1.**
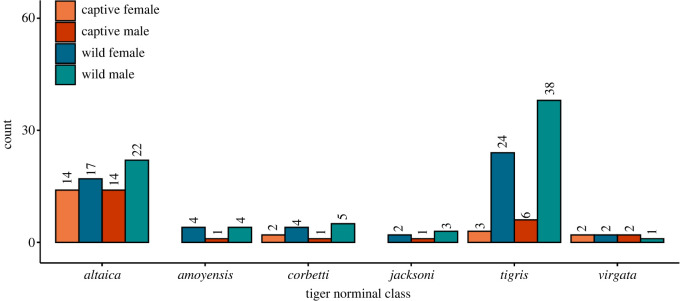
Available data for analysis grouped by sex, captivity status and by recorded putative subspecies for the continental tiger. In total, 98 males (25 captive, 73 wild) and 74 females (21 captive, 53 wild) were used in the analysis.

The dataset consists of considerably more wild specimens than captive specimens, the geographical origin of captive and wild specimens is not random, and certain putative taxonomic groups are represented in greater or lesser numbers depending on captivity status (figure 1). Captive tigers predominantly consisted of specimens recorded as *P. t. altaica* (Amur tigers), which were also well represented by wild specimens. A large number of wild tigers from the Indian subcontinent was available, but there were very few captive specimens from this group. The scarcity of tiger specimens in museum collections recorded as *amoyensis, corbetti, jacksoni* and *virgata* represent a geographical scarcity from China, continental Southeast Asia and the Caspian region. The available data allow three levels of analysis: (i) an analysis of all data, (ii) an analysis of male and female continental tigers separately, which provides two independent datasets for validation, and (iii) an analysis of groupings from similar geographical origins. Given the data available, captive and wild Amur tigers from the Russian Far East were analysed separately.

**Figure 2.**
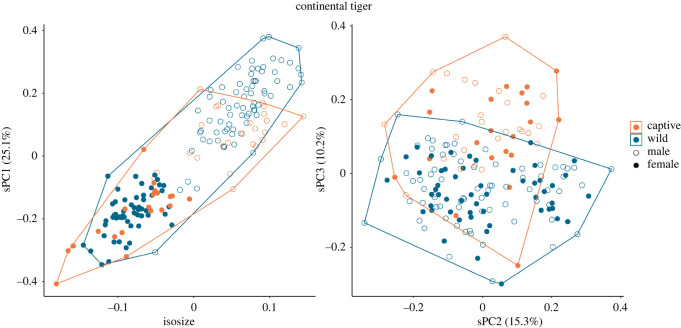
The relationship between captive and wild specimens by isosize and shape sPCs, when male and female continental tigers are analysed together. Male tigers separate from females in both size, and sPC1, which accounts for allometric scaling. sPC3 shows some differentiation by captivity status.

Considering male and female tigers together (figure 2), there is clear size-related sexual dimorphism, with males being larger and with minimal overlap between the sizes of the sexes. The correlation between shape principal component 1 (sPC1) (25.1% contribution) and isosize suggests this component accounts for allometric scaling. No separation by sex or captivity status is apparent in sPC2 (15.3% contribution), but there is some indication that captive individuals differ from wild individuals across sPC3 (10.2% contribution), although there is considerable overlap.

**Figure 3.**
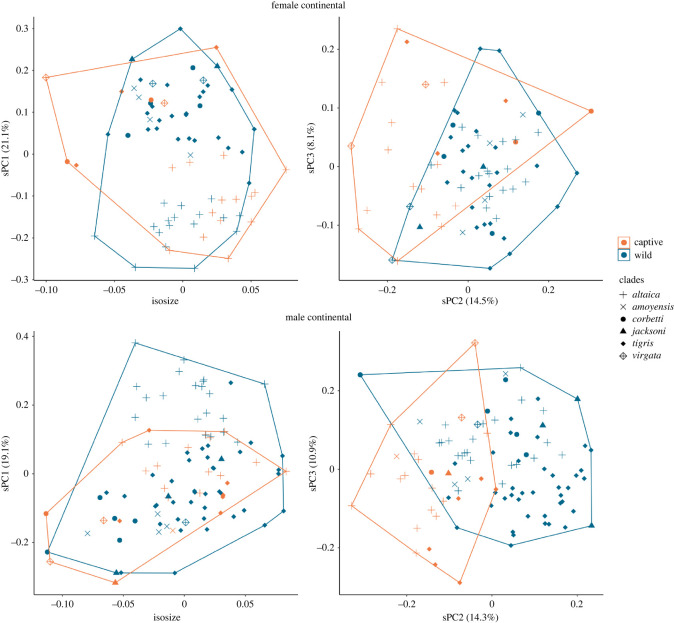
Comparison between skulls of captive and wild continental tigers by isosize and sPCs for each sex. The geographical origin of both captive and wild specimens is represented by genetic clade (*altaica* = Amur tigers from the Russian Far East, *amoyensis* = South China, *corbetti* = Indochinese, *jacksoni* = Malayan, *tigris* = Indian subcontinent, *virgata* = Caspian). sPC1 separates Amur tigers from other continental tigers, while sPC2 shows separation by captivity status.

When male and female tigers are considered separately (figure 3), the data show some differentiation by captivity status across sPC2 (14.3 and 14.5% contributions, respectively). sPC1 differentiates between putative subspecies classification, especially between Amur tigers and other continental tigers (19.1% and 21.1% contribution for male and female tigers, respectively). All other clades show considerable overlap in PCs of shape and size. Electronic supplementary material, figures S3–S5 display loadings for all sPCs.

**Figure 4.**
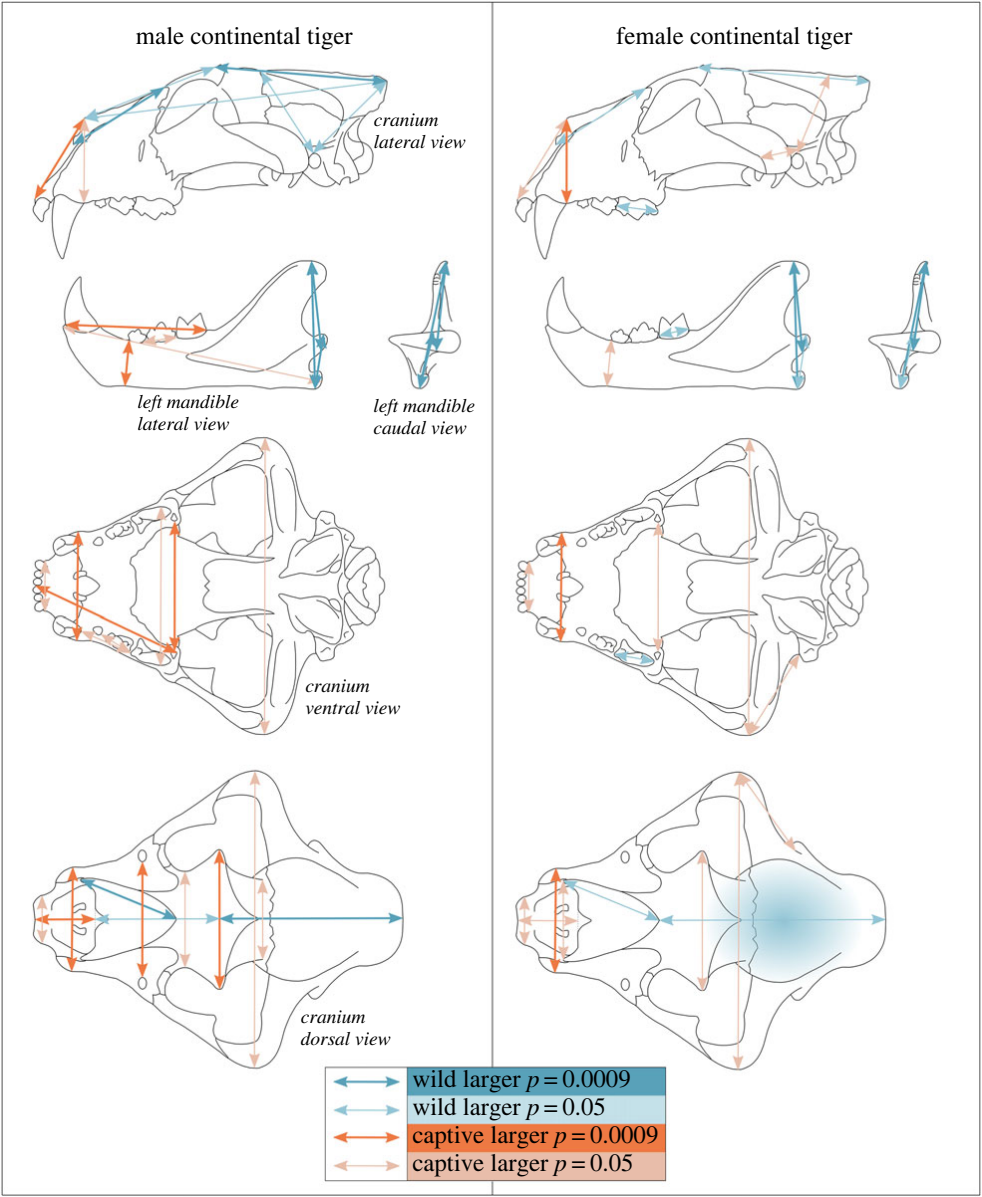
A graphical representation of measurements that are significantly different between captive and wild continental tigers. Cranial volume is represented by shading in the cranial region. Significance is determined by *t*-tests (electronic supplementary material, table S1) based on values of 0.05 and after a Bonferroni correction, 0.0009.

Measurements, which differ significantly between captive and wild specimens (determined by *t*-tests—electronic supplementary material, table S2), are shown in figure 4*.* They show broadly similar patterns between captive and wild specimens for each sex. Measurements of overall skull length are not consistently affected by captivity status, which is concordant with plots showing no differentiation between captivity status by isosize. Because the variables are scaled by isometric size, large measurements of skull length exhibit low variance. Rostral depth and breadth, and palate breadth measurements are generally larger in captive specimens. Anterior facial length is larger in captive specimens, whereas posterior facial length, nasal length and head length are smaller.

Measurements of the neurocranium show that, similar to the rostrum, skull breadth measurements are generally larger in captive specimens. Foramen magnum height is not significantly different between captive and wild continental tigers, and while cranial volume is on average larger in wild specimens, it only approaches significance in female tigers. Wild tigers have taller posterior mandibles, which surround the muscular insertion points of the coronoid and angular processes, yet anterior mandibular depth is broader in captive individuals. Tooth lengths are not consistently different between captive and wild specimens.

Measurements of skull length, orbit, facial length, palate-inion, overall zygomatic length and both cranial height and skull height are not consistently or significantly different between wild and captive tiger skulls. The postorbital bar does not differ between wild and captive tigers, but is of interest due to its relatively large variance in both captive and wild specimens (electronic supplementary material, figure S8).

**Figure 5.**
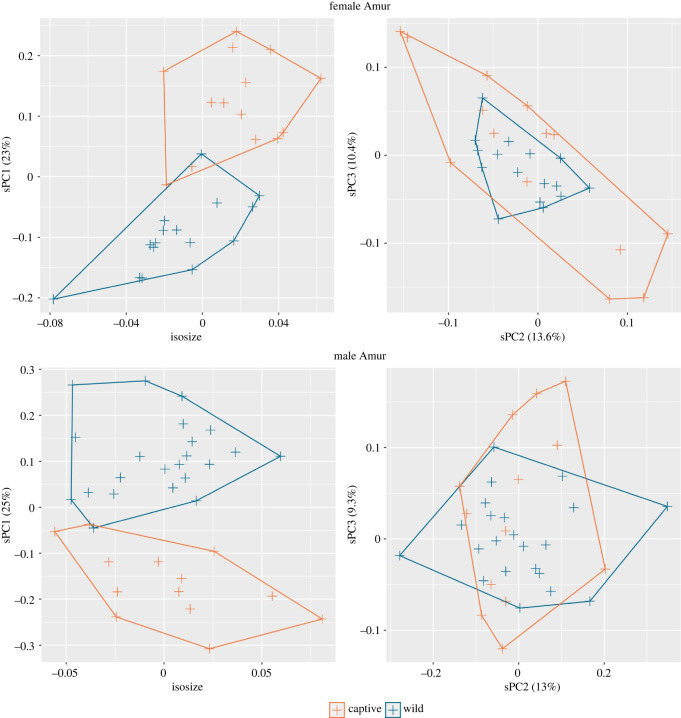
Comparison between captive and wild Amur tiger skulls by isosize and sPCs for each sex. When sex and geographical origin/clade are the same, the first PC of shape (sPC1) accounts for differences in captivity status.

There is variation in skull dimensions between the Amur tiger and all other continental tiger clades (figure 3). Differences between the Amur tiger and other continental tigers are shown through sPC1, with some overlap in males, and little overlap in females. The number of both captive and wild Amur tigers from the Russian Far East enables further analysis of captive and wild individuals from a single geographical origin (figure 5). Captivity status is distinguishable between specimens across sPC1 in female and male Amur tigers (23% and 25% contribution, respectively) with very low overlap. By using individuals from a single geographical origin, captivity status is the largest source of variation between individuals.

**Figure 6.**
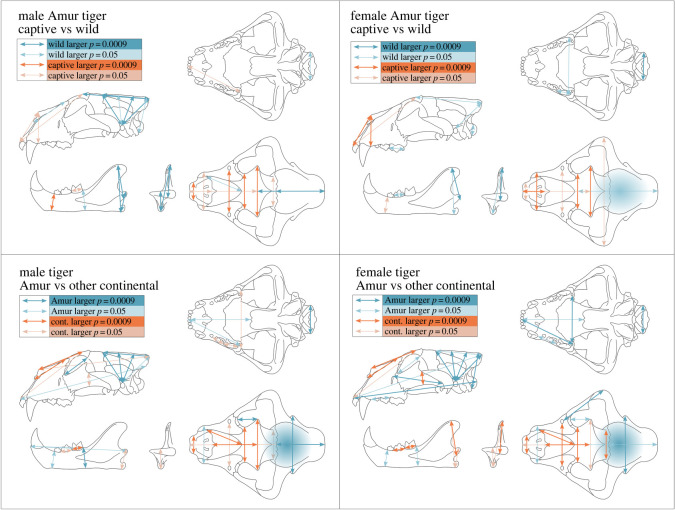
Variation of size-independent (scaled) variables by captivity status for the Amur tiger, and a comparison between wild Amur tigers and other wild continental tigers. Cranial volume is represented by shading in the cranial region. Measurements that differ significantly are highlighted by which population mean is larger. Significance is determined using *t*-tests (electronic supplementary material, table S1) based on values of 0.05 and after a Bonferroni correction, 0.0009.

The same broad pattern of variation between captive and wild continental tigers is found between captive and wild Amur tigers in both males and females (figure 6). Additionally, sagittal crest length and cranial height (which account for sagittal crest height) of male Amur tigers are significantly larger in wild specimens, which is not apparent in comparisons using all continental tigers. There is some visual suggestion that this pattern also occurs in female Amur tigers, but it is generally not significant.

Wild Amur tiger skulls exhibit morphological patterns, which are concordant with more ‘wild’ characteristics compared with other continental tigers (figure 6; electronic supplementary material, figures S11–S15). Wild Amur tigers have increased cranial volumes, cranial heights, longer sagittal crests, wider mastoid breadths, and narrower rostra and nasal widths when compared with other wild continental tigers. Conversely, wild female Amur tigers have reduced mandible heights, which are more similar to captive specimens. While the mandible height of the angular process is smaller in wild male Amur tigers than other wild continental tigers, it is not significant when accounting for the Bonferroni correction, and no significant difference is found in the mandible height of the coronoid process. The postorbital bar of wild Amur tigers is significantly smaller than that of other wild continental tigers. This measurement exhibits high variance, but has no discriminatory power between captive and wild specimens.

## 4. Discussion

There is clear shape differentiation, but no size difference, between the skulls of captive and wild continental tigers, which is consistent with previous studies in big cats [8]. Skull size between wild felids has not been found to correlate with variations in jaw mechanics [30], but it could be affected by nutrition. Beyond measures of overall size, the results highlight measurements that are not consistently affected by captivity across all regions of the skull. It is likely that these measurements are less affected by bite forces, such as measurements of the orbit and skull height. While it is possible that causes other than the mechanical properties of diet may have played a role in differentiating by captivity status, the lack of change in areas of the skull not affected by bite forces make this less likely. Hollister [42] found captive lions to have a thickened malar, which roughly corresponds to the measurement of the postorbital bar described here. While the results here show very large variance in the postorbital bar, as shown in the loadings of sPCs, it does not show any statistical difference between wild and captive tigers (electronic supplementary material, table S2). A low mean coefficient of variation shows that this measurement is robust to error (electronic supplementary material, table S1) and so variation is unlikely to have come from poor measurement accuracy.

When specimens of similar taxonomic status and geographical origins are analysed (Amur tigers), the differentiation between captive and wild specimens is markedly more apparent than when multiple geographic groups are analysed together. When Amur tigers are analysed separately, captivity status accounts for the first PC of shape, with very little overlap between captive and wild individuals in this axis. Captive Amur tigers represent similar genetic diversity, in terms of microsatellite composition and heterozygosity, to that of their wild counterparts, and both wild and captive populations exhibit low levels of inbreeding [22]. Therefore, morphological differences due to genetic variation between captive and wild populations are unlikely. While the use of *t*-tests has highlighted significant individual measurements, understanding the differences between captive and wild specimens is best approached by examining groups of measurements. This approach prevents the over-interpretation of the effects of individual measurements and provides a more holistic interpretation of skull differentiation. We do not explicitly address modularity and integration in the skulls of continental tigers here (e.g. [49,50]), yet patterns have emerged between measurements in close proximity to one another within discrete regions. The wider skull dimensions and shorter mandible heights of captive continental tigers have been found in previous studies and probably relate to the different mechanical properties of captive diets [8,42]. The primary jaw-closing muscles are the temporalis and masseter [30]. The masseter muscles originate on the zygomatic arch [8] and insert on the mandible at the angular process [51]. A high coronoid process improves the mechanical leverage of the temporalis muscle [30] for a more powerful bite, and it is therefore conceivable that variation in the degree of development of the temporalis muscle through usage may affect the height of the coronoid process during development. Stresses occur across the rostrum in big cats during twisting, shaking and biting [52], which have probably constrained anterior skull width and height in wild tigers, where generated forces are higher or more prolonged during killing of live prey. Skull shape differentiation is apparent here despite many European zoos using enrichment practices and partial/whole carcass feeding, which better replicate natural stresses on the skull and reduce stereotypical behaviours [53–55]. Beyond the mechanical effect of feeding on carcasses, the additional physical effort involved in prey capture, dragging carcasses into cover and manipulating them, including breaking through the tough skin of large ungulates, is not usually experienced by captive tigers [55]. Additionally, captive tigers probably have a lower dietary consumption rate than wild tigers, owing to their lower energy needs, and therefore spend less time feeding. Both of these factors reduce the time and effort required for feeding, and are likely to contribute to smaller masticatory muscle size and lower forces acting upon the skull in captive specimens.

There is clear separation of skull shape between Amur tigers and other continental tigers of a similar size. Previous craniometric analysis has shown the Amur tiger as the most distinct of the continental tiger populations, whilst Indian, Indochinese and South China tigers show clear morphological overlaps [17]. It would be expected that there would be a clinal relationship in the morphology of tigers from eastern Asia with latitude, if more specimens from mainland China were available. However, there are very few specimens available in museum collections to test this hypothesis. We find that the difference in skull parameters between wild and captive Amur tigers mirrors the differences between wild Amur tigers and other wild continental tigers. Wild Amur tigers have narrower rostra and greater cranial volumes than both Amur tigers in captivity and other wild continental tigers. The difference in cranial volume between wild tiger populations, which follows the same pattern as other captive–wild disparities, in part dispels the notion that differences in cranial volume in captivity are pathological in big cats [4]. Indeed, a reduction in cranial volumes of wild species bred in captivity is commonly seen in many taxa [7]. Additionally, wild Amur tigers have more pronounced sagittal crests (cranial height and sagittal crest measurements) than both captive Amur tigers and other wild continental tigers. Differentiation in the sagittal crest by captivity status in Amur tigers has been suggested by previous studies, which propose that a flattening of the sagittal crest is due to a reduction in the development of the temporalis [6]. The superficial attachment of the temporalis on wild tigers probably promotes the development of the sagittal crest, as an increased skull surface area is required for an increased temporalis mass when compared with less muscular captive tigers. Isometric scaling of the temporalis muscle mass requires an allometric increase in surface area for the muscle's origination, which is achieved by development of the sagittal crest. The lesser differentiation by captivity status on sagittal crest height or length in female Amur tigers is probably due to their generally less pronounced sagittal crest for a given body size regardless of captivity status.

Owing to the known skull plasticity found by comparisons between captive and wild Amur tigers, the increased cranial volume, cranial height, sagittal crest length, mastoid breadth, and decreased rostrum size of Amur tigers, compared with other continental tigers, is probably a plastic response to the vastly different environmental conditions of the Russian Far East, compared with the rest of the continental tiger range, which have promoted greater frequency and magnitude of forces acting on the skull and mandible in killing prey and chewing. The prey composition and preferences of the Amur population could have led to skull differences, yet large deer species, similar in body size to the red deer found in the Russian Far East, are preferentially preyed upon in southern continental tiger populations, and wild pigs, *Sus scrofa*, are a ubiquitous staple of tiger diets [19,56,57]. We propose two possible scenarios for increased jaw musculature in wild Amur tigers compared with other wild continental tigers that have resulted in the skull morphological changes reported here: Amur tigers may well have to chew through frozen carcasses in winter when they stay beside a kill until it is consumed, requiring increased masticatory forces when compared with other continental tigers. Amur tigers favour larger prey items during the winter compared with the summer months, consuming kills over several days [58], with average January temperatures in the Primorsky Krai averaging −11.6°C [59]. Alternatively, or acting in unison, Amur tigers consume more prey per day than continental tigers from southern populations due to increased energetic requirements from thermoregulatory demands [58] and are therefore using their jaw musculature more often. As tiger cubs begin consuming meat at around three months of age [60], differences in the quantity of consumed prey, and/or the mechanical properties of carcasses, will affect tigers during their development and into adulthood. Caspian tigers, which are phylogenetically closest to Amur tigers [12,61] and yet are morphologically more similar to other continental tigers (although the sample of Caspian tigers is very small), would have been subjected to milder winter conditions than those of the Russian Far East and therefore not subjected to the same energy demands or frozen food sources. Whilst differences in skull shape between Amur and other continental tigers are similar to differences between the skulls of captive and wild specimens, it should be noted that mandible heights in female Amur tigers are smaller than in other continental tigers, which contradicts this trend. While we have shown that the height of the coronoid process increases with increased masticatory forces through our captive–wild comparison, this highlights a potential genetic component in differentiating the skulls of Amur tigers from other continental tigers made possible through the geographical dissociation of the Amur population from other continental tigers since approximately 8 ka [14]. Or this possible genetic difference may have resulted from the population bottleneck, which occurred in the Amur tiger population in the early twentieth century. It is possible that in addition to environmental and dietary differences, the increased height of the sagittal crest in female Amur tigers, which we have shown to be phenotypically plastic, is compensatory for what is likely to be reduced mechanical advantage of the mandible. However, this hypothesis requires further testing of mechanical advantage using three-dimensional geometric morphometric techniques and moment-arm analyses which address muscle attachment sites.

## 5. Conclusion

The results presented here show that the skulls of male and female continental tigers differ between wild and captive environments. While overall skull size does not differ between wild and captive specimens, an overall pattern of wider skull dimensions and shorter mandible heights is found in captivity. The results highlight the importance of comparing specimens from comparable taxonomic or geographical origins, as morphological differences across the geographical range of a species can obscure patterns between captive and wild specimens. When similar geographical groupings are analysed, there is minimal overlap between captive and wild specimens within the first PCs of shape.

The distinctive shape and characters of wild Amur tiger skulls that differentiate them from the skulls of continental tigers would seem to support their recognition as a distinct subspecies. However, wild and captive Amur tigers differ predominantly by skull parameters associated with forces acting on the skull and mandible during killing of prey and feeding, which closely match differences between wild Amur tigers and other continental tigers. This suggests that phenotypic plasticity is the main driver of differentiation between wild Amur tigers and other continental tigers. It is likely that the vastly different environmental conditions of their now isolated range reflect most of their morphological distinctiveness, rather than differentiation through evolutionary divergence, which has occurred only recently during the Holocene.

The results here point to the value of using captive-bred animals for comparison with wild animals, in order to determine which skull characters vary due to phenotypic plasticity or genetic determination. This kind of analysis allows for a careful differentiation between characteristics that are due to phenotypic plasticity and hence of no taxonomic value, such as sagittal crest size, from those that are likely to reflect a taxon's evolutionary history. This may also help resolve disparities between genetic and morphological studies, and demonstrate the degree of adaptability of species which have very large global distributions.

## Data Availability

The data used here is available through the Dryad Digital Repository: https://doi.org/10.5061/dryad.pk0p2ngs1 [62]. Supplementary material is available online [63].
